# Review of the Recent Changes in the WHO Classification for Pediatric Brain and Spinal Cord Tumors

**DOI:** 10.1159/000528957

**Published:** 2023-01-06

**Authors:** Aaron M. Halfpenny, Matthew D. Wood

**Affiliations:** Department of Pathology and Laboratory Medicine, Oregon Health & Science University, Portland, Oregon, USA

**Keywords:** Central nervous system neoplasms, Neuropathology, Pediatric brain tumors, WHO classification

## Abstract

**Background:**

Periodic updates to the World Health Organization (WHO) classification system for central nervous system (CNS) tumors reflect advances in the pathological diagnosis, categorization, and molecular underpinnings of primary brain, spinal cord, and peripheral nerve tumors. The 5th edition of the WHO Classification of CNS Tumors was published in 2021. This review discusses the guiding principles of the revision, introduces the more common new diagnostic entities, and describes tumor classification and nomenclature changes that are relevant for pediatric neurological surgeons.

**Summary:**

Revisions to the WHO CNS tumor classification system introduced new diagnostic entities, restructured and renamed other entities with particular impact in the diffuse gliomas and CNS embryonal tumors, and expanded the requirements for incorporating both molecular and histological features of CNS tumors into a unified integrated diagnosis. Many of the new diagnostic entities occur at least occasionally in pediatric patients and will thus be encountered by pediatric neurosurgeons. New nomenclature impacts the terminology that is applied in communication between pathologists, surgeons, clinicians, and patients. Requirements for molecular information in tumor diagnosis are expected to refine diagnostic categories while also introducing practical considerations for intraoperative consultation, preliminary histological evaluation, and triaging of neurosurgical tissue samples for histology, molecular testing, and clinical trial requirements.

**Key Messages:**

Pediatric brain tumor diagnosis and clinical management are a multidisciplinary effort that is rapidly advancing in the molecular era. Interdisciplinary collaboration is critical for providing the best care for pediatric CNS tumor patients. Pediatric neurosurgeons and their local neuropathologists and neuro-oncologists must work collaboratively to put the most current CNS tumor diagnostic guidelines into standard practice.

## Introduction

The 5th edition of the World Health Organization (WHO) Classification of Central Nervous System (CNS) Tumors (WHO CNS 5) was published in electronic form in November 2021 and in print in February 2022 [1]. This provided updates to the systems of diagnosis and classification for brain, spine, and peripheral nerve tumors, reflecting advancements in the understanding of the molecular underpinning for these neoplasms, recognition of the histological diversity within existing and novel diagnostic entities, and unified CNS tumor nomenclature and reporting structure with other organ systems [2].

In this review, we summarize the neuropathological diagnostic updates that are most relevant for pediatric neurosurgeons. Our goals are to (1) provide a solid foundation on the major overarching principles that guided WHO CNS 5, (2) communicate changes in neuropathology nomenclature, diagnostic categories, and reporting that neurosurgeons will encounter in their practice, and (3) introduce the new glial, glioneuronal, and embryonal tumor diagnostic entities that are most relevant in pediatric patients. In total, 22 newly recognized tumor types are included in WHO CNS 5, and 13 other tumor types have updated nomenclature and/or categorization [2]. Because this review aims for breadth, readers are referred to other recent reviews for further details on focused topics including an overview of the WHO CNS 5 [2], ependymal tumors [3], diffuse gliomas [4], embryonal tumors [5, 6], low-grade glioneuronal tumors [7], diagnostic reporting structure [8], and treatment considerations [9, 10]. Mesenchymal (both meningothelial and non-meningothelial), cranial/paraspinal nerve, hematolymphoid, germ cell, pituitary, pineal, choroid plexus, and melanocytic CNS tumors are not reviewed here.

## Updates in Diagnostic Reporting

WHO CNS 5 expands the use of molecular information for brain tumor diagnosis, classification, and grading. Molecular features are put in context with tumor histology, leading to an *integrated diagnosis* that incorporates both elements into the final classification and grade. The WHO and the International Society for Neuropathology endorse a layered reporting system that separates the integrated diagnosis from the histological classification, tumor grade, and molecular information [2, 8]. This allows for precise communication of pathological information and makes explicit the supporting findings upon which an integrated diagnosis rests (examples in Table 1). Tumor grades are now given with Arabic numerals instead of Roman numerals to reduce the possibility of typographical or interpretive errors and to bring the CNS classification in line with WHO grade reporting in other organ systems. Intrinsic brain and spine tumors are specifically designated as “CNS WHO grade 1/2/3/4” to distinguish the CNS system from other organ systems-based WHO classifications.

The requirement for molecular information in tumor diagnosis presents a challenge if the testing is not available or if there are technical limitations due to the quality or amount of tissue. If necessary molecular information is unavailable or cannot be obtained, tumors are designated “Not Otherwise Specified (NOS)” [11]. If molecular information is available but a tumor does not align with a specific WHO CNS 5 entity, the designation “Not Elsewhere Classified (NEC)” is applied instead [11]. A helpful point of reference for pathologists, neurosurgeons, and other clinicians is the introduction of *essential* and *desirable* diagnostic criteria for each tumor type. This gives succinct, summative guidance on the key features that are necessary for (or supportive of) a particular diagnosis.

## Changes in Tumor Categorization and Nomenclature

In a major restructuring, WHO CNS 5 separates the diffuse gliomas into three categories: (1) adult-type diffuse gliomas, (2) pediatric-type diffuse low-grade gliomas, and (3) pediatric-type diffuse high-grade gliomas. While category (1) is beyond the scope of this review, it is understood that there are no firm age cutoffs for either the adult-type or pediatric-type tumors. For example, isocitrate dehydrogenase (IDH) mutant diffuse gliomas are not exclusive to adult patients, and conversely, some diffuse gliomas in adults can have pediatric-type molecular features [12, 13].

CNS tumor grades are now assigned within each tumor type instead of considering each grade of tumor to be a separate subtype. Because of this change to “grading within types,” the diagnostic prefix of “anaplastic” has been removed. For example, in the previous revised 4th edition of the WHO classification system from 2016 (WHO CNS 4), “pleomorphic xanthoastrocytoma, WHO grade II,” and “anaplastic pleomorphic xanthoastrocytoma, WHO grade III” were considered two different entities. Now, these are unified under a single diagnostic category of “pleomorphic xanthoastrocytoma” with two different grades, CNS WHO grade 2 or 3.

The term “glioblastoma” is no longer used for pediatric tumors because this diagnosis now refers specifically to an adult-type diffuse glioma that is negative for IDH and H3 gene mutations and shows other defining histological and/or molecular changes [2]. The embryonal tumors have been further subdivided and refined with additional molecularly defined tumor types. The term “hemangiopericytoma” is superseded by “solitary fibrous tumor” due to the recognition of a shared molecular driver and to align the nomenclature for intracranial cases with systemic cases. A few distinct diagnostic entities that appeared in earlier WHO CNS editions have been absorbed into more general diagnostic categories, and some entities have been updated or have revised grades [2]. The relevant changes for pediatric tumors are described in subsequent sections and summarized in Table 2.

## Practical Considerations for Molecular Profiling

Pediatric CNS tumors show a range of genetic alterations including DNA sequence alterations, gene rearrangements, and gene amplifications/deletions. Depending on the entity, a precise diagnosis might require evidence for specific genetic alterations. The specific testing methods are up to each institution and subject to testing availability. WHO CNS 5 recognizes a role for epigenetic subgrouping of CNS tumors by genome-wide DNA methylation-based profiling (DNA-MP). This technology has potential to refine tumor diagnosis and to identify new, clinically relevant tumor types and subtypes [14, 15]. However, DNA-MP has limited availability, and there are considerations for billing and reimbursement that have not yet been addressed in the USA. A compatible tumor categorization by DNA-MP is currently a desirable − but not essential − criteria for most of the WHO CNS 5 diagnostic entities. It is one of the essential criteria for the diagnosis of two novel entities that are discussed below: diffuse glioneuronal tumor with oligodendroglioma-like features and nuclear clusters (DGONC) and high-grade astrocytoma with piloid features (HGAP).

The increasing reliance on molecular information in modern diagnosis reflects heterogeneity in pediatric brain and spine tumor histologic, radiologic, and clinical features. This requirement for molecular information limits the diagnostic information that can be provided at the time of intraoperative consultations. Depending on the clinical and radiologic context and with only a limited sampling of frozen tissue (where microscopic features may be sub-optimal at best or misleading at worst), it may only be possible to give a general categorization for a primary CNS tumor based on histological resemblance to a cell of origin and growth pattern, with deferral to permanent sections and ancillary testing for a specific diagnosis and grade. Tissue that is used for intraoperative consultation can be depleted or altered in the process of a frozen section, rendering the sample sub-optimal or unusable for further immunohistochemical and molecular studies. Therefore, intraoperative consultations that will not alter the operative or immediate post-operative management should be avoided given the increasing need for ancillary studies which may be compromised by sample size and previous freezing of the tissue. Clear communication between surgeons and pathologists is required, including a mutual understanding of the indications for an intraoperative consultation, clear expectations from both parties on what meaningful information can be obtained, and appreciation of the risks of extensive tissue sampling and limitations of intraoperative histological analysis.

Pediatric CNS tumor diagnosis and molecular testing of tumor tissue can suggest the possibility of an underlying genetic syndrome such as neurofibromatosis type 1 or type 2, rhabdoid tumor predisposition syndrome, DNA replication/repair deficiency syndromes, Gorlin syndrome, Cowden syndrome, tuberous sclerosis complex, Li-Fraumeni syndrome, and many others. The possibility of a germline tumor syndrome should be considered clinically in the setting of any new pediatric brain tumor diagnosis, and constitutional genetic testing may be indicated.

### New Glioneuronal and Neuronal Tumors

Tumors in this diagnostic category show varying degrees of morphologic and/or immunophenotypic evidence of neuronal or glial and neuronal differentiation. This includes previously defined, commonly pediatric entities of ganglioglioma, desmoplastic infantile ganglioglioma/astrocytoma, dysembryoplastic neuroepithelial tumor, rosette-forming glioneuronal tumor, diffuse leptomeningeal glioneuronal tumor, and other tumors [7]. The three new tumor types in this category are myxoid glioneuronal tumor (MGNT), multinodular and vacuolating neuronal tumor (MVNT), and DGONC.

**Fig. 1:**
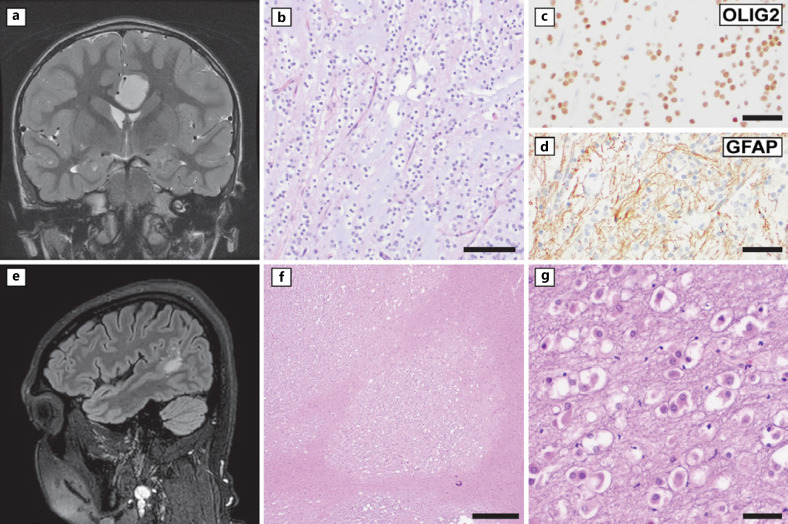
New entities within neuronal and glioneuronal tumors. **a–d** Example of a myxoid glioneuronal tumor (MGNT) presenting as a left frontal parasagittal mass in a 5-year-old boy with seizures. **a** T2-weighted imaging showed a 2.5 cm T2-hyperintense circumscribed lesion with mass effect on the corpus callosum and adjacent brain parenchyma, without contrast enhancement (not pictured). **b** Histology shows tumor cells with monomorphous, rounded nuclei and clear perinuclear spaces with delicate background branching capillaries and a rich matrix of purple-staining mucinous (myxoid) material. **c, d** The tumor showed strong immunoreactivity for glial markers OLIG2 and glial fibrillary acidic protein (GFAP). **e–g** Examples of multinodular and vacuolating neuronal tumor (MVNT). **e** T2-FLAIR imaging from a 32-year-old man with seizures shows punctate subcortical white matter signal. **f, g** Histological features in MVNT include nodular patterning of tumor cells clustered within white matter and cells with neuronal features sitting in small, non-mucinous cystic spaces. Scale bars, 100 microns (**b**), 50 microns (**c, d, g**), and 500 microns (**f**).

MGNT, shown in Figure 1a-d, is a CNS WHO grade 1 tumor most often found in the region of the septum pellucidum and corpus callosum and rarely in the lateral periventricular white matter [16]. The majority of patients are under 30 years of age, with range as young as 6 and as old as 65 years [17, 18]. Radiologically, MGNT is usually T1-hypointense, T2-hyperintense, without contrast enhancement or restricted diffusion, and lacking calcifications [17]. Intraoperative gross findings are of a markedly soft, gelatinous, gray mass. Microscopically, MGNT is mostly non-infiltrative and consists of oligodendrocyte-like cells within an abundant myxoid (mucinous) background, accompanied by delicate thin-walled capillaries. Floating neurons, neuropil, and neurocytic rosettes may be present [18]. At its discovery, MGNT was set apart from other known glial/glioneuronal tumors by its location and identification of a distinct genetic driver − a dinucleotide substitution in the extracellular domain of the *PDGFRA* oncogene leading to a lysine (K) to leucine (L) or isoleucine (I) amino acid substitution at position 385 (p.K385L/p.K385I) [19]. Further studies showed that MGNT has a distinct epigenetic signature by DNA-MP. Thus, identification of a *PDGFRA* p.K385L/I mutation, certain other less common *PDGFRA* alterations, and/or a methylation-based subgrouping of MGNT can support the diagnosis. Befitting its low proliferative index and CNS WHO grade 1 designation, MGNT has a favorable prognosis in the small number of cases identified to date. In one series of 38 patients, there were no deaths as a result of disease [17]. Tumors may recur locally or occasionally show ventricular dissemination; however, in the limited data available at this time, such cases appear to still be associated with favorable long-term outcomes [17, 18]. The radiologic, demographic, and histological spectrum of MGNT is likely to expand as more cases are identified.

MVNT, shown in Figure 1e-g, is an epilepsy-associated CNS WHO grade 1 neoplasm seen occasionally in the pediatric age group, with ∼15% of cases undergoing surgery in the first or second decade of life [1]. This tumor shows radiologically characteristic clustered T2-FLAIR-hyperintense nodules in deep cortex and subcortical white matter and arises most commonly in the temporal lobes (Fig. 1e) [20, 21]. Histology shows discrete nodules of hypomyelinated white matter containing moderately cellular neuronal-like tumor cells resting in small, non-mucinous vacuolar spaces (Fig. 1f, g). A variety of mitogen-activated protein kinase (MAPK) pathway alterations are reported, with small activating insertions/deletions of the *MAP2K1* gene being common [22]. The characteristic radiologic findings and low-grade designation suggest that some patients may be monitored radiologically without resection in the appropriate setting [23].

DGONC is a provisional entity with a median presenting age of 9 years [24, 25]. A recurrent genetic driver has not been identified to date, but loss of chromosome 14 is common and can suggest the diagnosis. Recognition of this tumor can be challenging because of high heterogeneity and frequent unusual microscopic features. In retrospective studies, the grading of these tumors has ranged from WHO 1 through 4, with a wide range of histological diagnosis and frequent unspecific descriptive diagnoses [24]. To date, all cases are supratentorial and occur most commonly in the temporal lobes [24]. Recurrent radiologic findings are not well described at this time. Typical histology shows a moderately to highly cellular tumor with infiltrative, predominantly oligodendroglial-like cells and scattered multinucleate cells with nuclear clusters that may show pleomorphism [1]. Due to the lack of a known genetic driver and the histological heterogeneity of these cases, WHO CNS 5 requires a compatible DNA methylation profile along with supportive morphology and immunophenotype [1]. There is minimal data on treatment implications for outcomes, but reported 5-year progression-free survival rate is 79% and 5-year overall survival rate is 86% in 12 patients with available follow-up [24]. A specific CNS WHO grade is not assigned at this time, and further outcomes studies are needed for this rare entity [24].

### New Circumscribed Astrocytic Gliomas

**Fig. 2:**
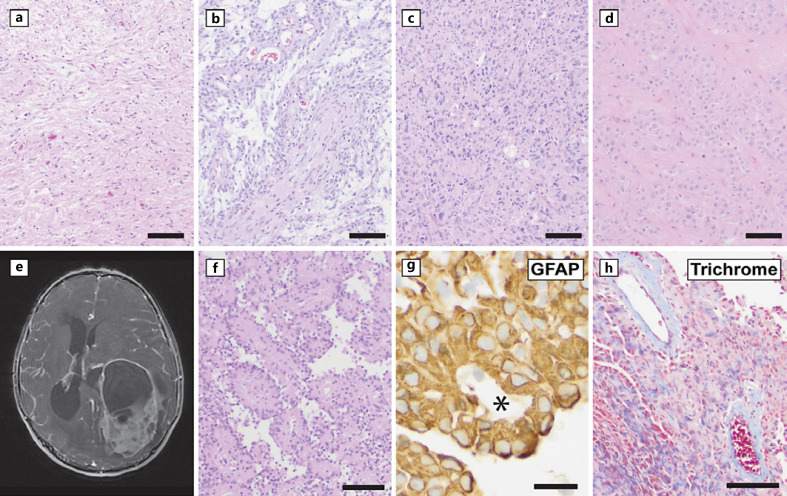
Circumscribed astrocytic gliomas. **a–d** The circumscribed astrocytic gliomas include PA (**a**) and its subtype pilomyxoid astrocytoma (**b**), pleomorphic xanthoastrocytoma (**c**), and subependymal giant cell astrocytoma (**d**). Note prominent, bright pink Rosenthal fibers in (**a**), which are not typically seen in pilomyxoid astrocytoma (**b**). **e–h** Astroblastoma, *MN1*-altered, is an updated entity in the category of circumscribed astrocytic gliomas. **e** In this example case, neuroimaging from a 6-year-old female with seizures showed a complex, cystic, heterogeneously enhancing parietal region mass (T1 post-contrast). Histology showed a glioma with a papillary growth pattern (**f**) and tumor cells with thickened radiating processes encircling blood vessels (asterisk) highlighted by GFAP immunohistochemistry (**g**). Perivascular hyalinization, highlighted by blue staining with trichrome is another supporting histologic feature (**h**). Scale bars, 50 microns (**a–d**), 100 microns (**f, h**) and 20 microns (**g**).

This group of gliomas is characterized by relatively compact, non-infiltrative tumor growth. Included in this category are the established entities of pilocytic astrocytoma (PA), pleomorphic xanthoastrocytoma, subependymal giant cell astrocytoma, and chordoid glioma, all of which remain largely unchanged in the WHO CNS 5 except for an expanded role for genotypic and histological correlation and the introduction of DNA-MP as a desirable diagnostic criteria (examples shown in Fig. 2a, c, d). Pilomyxoid astrocytoma (PMA − Fig. 2b) is classified as a subtype of PA that is distinguished histologically by a prominent perivascular arrangement of tumor cells, a mucinous (myxoid) background, and increased cellularity, and clinically by its more common occurrence as a hypothalamic or optic chiasm mass in infants. Pilomyxoid astrocytoma can potentially show more aggressive clinical behavior compared to classical PA including local recurrence and cerebrospinal fluid dissemination; however, it is not assigned a higher grade at this time [1]. HGAP is a newly introduced circumscribed astrocytic glioma, and the entity of astroblastoma from prior WHO CNS has been revised to include genetic information.

Astroblastoma was defined histologically in previous WHO CNS editions, but there is now recognition that astroblastoma-like features can occur in other tumor types such as ependymoma and pleomorphic xanthoastrocytoma [26, 27]. Rearrangements involving the *MN1* gene with common partners being *BEND2* and *CXXC5* are a defining genetic feature that is essential for the diagnosis in WHO CNS 5 [1]. Patients present as young as 3 years old with a median age of 15 years, and there is a very pronounced female bias [28]. Typical imaging findings are that of a circumscribed cerebral hemispheric (rarely brain stem or spinal) mass with heterogenous contrast enhancement, adjacent edema, and occasional cystic changes, as shown in an example in Figure 2e-h [29, 30]. Histologically, the tumor is well-demarcated from adjacent brain and includes radially arrayed glial cells forming perivascular pseudorosettes with stout or thickened processes termed “astroblastic pseudorosettes,” usually accompanied by perivascular fibrosis. Of note, these histologic findings are not universally present, and tumor can show a wide range of other features including rhabdoid cells and poorly differentiated embryonal-like components. There is histological overlap between astroblastoma and supratentorial ependymomas, and genetic studies may be required to resolve this differential diagnosis. Astroblastoma *MN1*-altered has limited outcome data, and there is no CNS WHO grade assignment at this time. Local recurrences appear to be frequent, but disease-specific survival appears favorable [26, 27].

HGAP is a diagnostically challenging tumor that may have PA-like or diffuse high-grade glioma-like histological features [31]. It is rare but probably under-recognized, with only 60 cases in the largest published series and 10% of those cases occurring in the pediatric age group. Due to the lack of specific histological or DNA sequence alterations and unspecific histologic features, DNA methylation-based classification is required for the diagnosis. Mutations of the *ATRX* gene (which can be inferred from an immunohistochemical marker), homozygous deletion or mutation of *CDKN2A/*B, and *NF1* or *BRAF* alterations are common [31].

## Updated Classification of Ependymal Tumors

Ependymomas have undergone significant reclassification according to anatomic site, histomorphology, and molecular findings, with 3 defined types occurring in each of 3 CNS anatomical compartments: supratentorial, posterior fossa, or spinal (9 total). Histological types of subependymoma and myxopapillary ependymoma are maintained in WHO CNS 5, while clear cell, tanycytic, and papillary ependymomas have been absorbed as morphologic variants of traditional ependymoma [32]. Subependymoma is designated CNS WHO grade 1 and myxopapillary ependymoma is designated CNS WHO grade 2. In any anatomical compartment, ependymomas other than subependymoma and myxopapillary ependymoma can be assigned a histologic grade 2 or 3, but the qualifier of “anaplastic” has been removed. If molecular information is not available, ependymomas are classified by anatomic location and labeled not otherwise specified (NOS). Since these tumors are circumscribed and gross total resection is associated with a better clinical outcome, recognition of an ependymoma on intraoperative consultation is important in guiding surgical management.

**Fig. 3:**
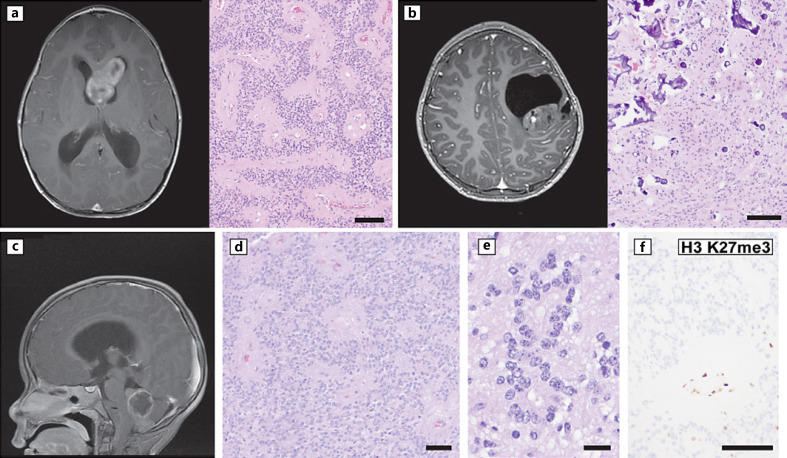
Ependymal tumors. **a, b** Examples of radiology and histology of supratentorial ependymoma, *ZFTA* fusion positive. **a** T1 post-contrast imaging and histology from a 5-year-old with headaches, nausea, and vomiting. The tumor showed well-formed perivascular pseudorosettes, represented at low power by the nucleus-free eosinophilic zones around small blood vessels. **b** T1 post-contrast imaging and histology from a 9-year-old with right-sided weakness showed a hemispheric mass presenting as a cyst with an enhancing nodule. Histological examination showed a cytologically bland tumor with extensive microcalcifications. **c–f** Example of posterior fossa group A (PFA) ependymoma. **c** T1-weighted post-contrast imaging from a 3-year-old with intractable vomiting, lethargy, and gait ataxia showed a posterior fossa mass. **d**−**f** Histological examination showed a non-infiltrative glioma with perivascular pseudorosettes (**d**, note similarity to panel **a**) and, on high-power examination, true ependymal rosettes (**e**). **f** Immunohistochemistry for trimethylated H3 K27 (H3 K27me3) shows loss of staining from tumor nuclei, consistent with PFA. Scale bars, 100 microns (**a, b, d**), 20 microns (**e**), and 50 microns (**f**).

Supratentorial ependymomas are divided into subependymoma (rare in children) and ependymomas with fusions involving *ZFTA* (an update to a pre-existing entity) or *YAP1* (a new entity added for WHO CNS 5). The legacy nomenclature of *RELA* fusion-positive ependymomas that appeared in WHO CNS 4 has been updated based on evidence that the fusion partner gene *ZFTA* (previously called *C11ORF95*) is more recurrent in this entity and can be rearranged with partners other than *RELA*. *ZFTA* fusion ependymomas are more common than *YAP1* and typically occur in the frontal and parietal lobes, with a median age of approximately 6.5 years at the time of surgery [33, 34]. Examples are shown in Figure 3a, b. This diagnosis requires evidence of *ZFTA* rearrangement and/or a compatible DNA methylation-based subgrouping along with compatible histology [1]. *CDKN2A* deletion in *ZFTA* fusion ependymomas has been associated with worse outcome [35]. *YAP1* fusion ependymomas account for <10% of supratentorial ependymomas, are more common in females, and occur at a younger age [1]. Available data from retrospective analysis suggest a more favorable outcome for *YAP1* fusion supratentorial ependymomas compared to *ZFTA* [1].

Posterior fossa ependymomas are separated by histology into subependymoma and traditional ependymoma, with the latter category further substratified into posterior fossa groups A (PFA) and B (PFB) ependymoma types. PFA is more common in infants and children and has a more aggressive clinical course (example shown in Fig. 3c-e). Tumor morphology does not distinguish between PFA and PFB, and definitive classification requires molecular studies. Loss of immunoreactivity for the trimethylated form of histone 3 lysine 27 (H3 K27me3) is a useful surrogate marker for most cases of PFA, and this feature can be helpful for provisional classification while molecular studies such as DNA methylation profiling are in process (Fig. 3f) [36]. Chromosome arm 1q copy number gain is associated with poor outcome in posterior fossa ependymomas, with some data indicating prognostic significance for PFA but not for PFB [37, 38]. Future work may further refine prognostic subgroups in posterior fossa ependymomas [39].

The spinal ependymomas include subependymoma (CNS WHO grade 1) and myxopapillary ependymoma, now designated CNS WHO grade 2 reflecting the potential for clinical behavior akin to traditional ependymoma. Traditional spinal ependymomas are less common in children and adolescents compared to adults, and when they do occur an evaluation for neurofibromatosis type 2 could be indicated. Rare spinal ependymomas have amplification of the *MYCN* oncogene and an aggressive clinical course. Most of the identified cases have been in adults, with rare examples in adolescence and none reported to date in infants or children [1].

## Introduction of Pediatric-Type Diffuse Low-Grade Gliomas

Pediatric-type diffuse low-grade gliomas are histologically diverse with astrocytic, oligodendroglial, or mixed/ambiguous histologic features, and have a wide range of molecular findings that mostly converge on activation of growth-promoting intracellular signaling pathways [40]. Angiocentric glioma was included in the previous WHO CNS 4 and is now recognized to have a characteristic *MYB::QKI* gene fusion in nearly all cases. Two newly defined CNS WHO grade 1 entities in this category are diffuse astrocytoma, *MYB*- or *MYBL1*-altered, and polymorphous low-grade neuroepithelial tumor of the young (PLNTY). A third, more general category of “diffuse low-grade glioma, MAPK pathway-altered” without a precise CNS WHO grade can be applied to low-grade diffuse gliomas (oligodendroglial or astrocytic) which do not fit into a specific category but are proven to have an activating alteration of the MAPK pathway without mutations of *IDH1*, *IDH2*, or H3 encoding genes or deletion of *CDKN2A* [1, 41]. Outcome is generally favorable and depends on tumor location, extent of resection, and genetic underpinnings [41]. Future studies on this group of tumors could identify more precise and clinically and pathologically distinct tumor subtypes.

**Fig. 4:**
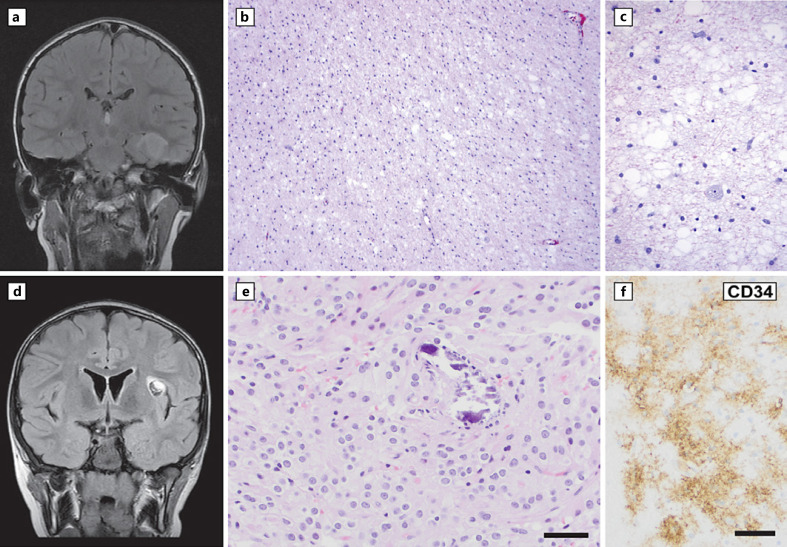
Pediatric-type diffuse low-grade gliomas. **a–c** Example of pediatric-type diffuse low-grade glioma, *MYB/MYBL1*-altered. A 9-year-old male patient with headaches was found to have a 3.6 cm left medial temporal lobe lesion, shown here on T2-FLAIR imaging. **b**−**c** Sections showed an infiltrative astrocytic glioma with microcystic and slightly myxoid background. **d–f** Example of polymorphous low-grade neuroepithelial tumor of the young. Neuroimaging studies from a 7-year-old boy with new-onset seizures showed a 1.3 cm left insular T2 intense cystic lesion (**d**, T2-FLAIR). Histology from the resection specimen showed oligodendroglial-like cells with scattered microcalcifications (**e**). Neoplastic ganglion-like cells and eosinophilic granular bodies were not present, and the tumor lacked the mucinous/myxoid background of DNET and MGNT. Strong extravascular labeling for CD34 (**f**) further supported the diagnosis. Scale bars, 50 microns (**e**−**f**), not available for panels B and **c**. DNET, dysembryoplastic neuroepithelial tumor. MGNT, myxoid glioneuronal tumor.

Diffuse astrocytoma, *MYB*- or *MYBL1*-altered (CNS WHO grade 1) accounts for about 2% of all pediatric low-grade gliomas, occurring mostly in the cerebral hemispheres or rarely in the brain stem in patients as young as age 4 [1]. There is a strong association with longstanding/refractory epilepsy. As demonstrated in Figure 4a-c, the tumor typically shows monomorphous, low-grade histology with astrocytic features, an infiltrative growth pattern, and microcystic spaces. The defining genetic alteration is a truncation or structural rearrangement of the *MYB* or *MYBL1* gene. The most common *MYB/MYBL1* partner genes are *PCDHGA1*, *MMP16*, and *MAML2* [42, 43]. The distinct driver sets this entity apart from adult-type diffuse astrocytic gliomas, other pediatric-type tumors that are driven by other MAPK pathway alterations, and from angiocentric glioma which has distinct histology and commonly shows the *MYB::QKI* fusion [42]. Prognosis is favorable, and improvements in seizure activity are expected with surgical intervention.

PLNTY was identified in 2017 upon review of a large cohort of epilepsy-associated brain tumors [44]. Available data suggest a slight female predominance and a broad age range, with median age at resection in mid-adolescence. Radiographic features overlap with other low-grade epilepsy-associated tumors, commonly with a cortical/subcortical location of a circumscribed, sometimes cystic mass with intratumoral calcifications and a predilection for the temporal lobes (example in Fig. 4d) [45]. As implied by the name of this tumor, PLNTY histology is highly variable. The most common histology is an infiltrative tumor with oligodendroglioma-like cells as shown in Figure 4e, accompanied by strong, diffuse cluster of differentiation 34 (CD34) immunoreactivity (Fig. 4f), the latter feature setting this entity apart from other diffuse pediatric-type low-grade gliomas [44]. A spectrum of driving alterations in the MAPK pathway are described, including *FGFR2* fusions to partner genes including *CTNNA3, INA*, and *KIAA1598* (among others) and *BRAF* mutation or fusion. Epigenetic analysis supports that PLNTY is a distinct entity [44, 46]. In a recent study, low-grade neuroepithelial tumors with *FGFR2* fusions aligned with PLNTY by epigenetic clustering even when their histologic features suggested ganglioglioma, MVNT, or were histologically unclassifiable. This underscores the broad histologic spectrum for PLNTY and demonstrates the utility of combining histological, genetic, and epigenetic approaches for identification [46]. The clinical outcome of PLNTY is favorable. Malignant transformation at recurrence of a tumor with initial histological features of PLNTY has been reported in one case where an *FGFR3-TACC3* fusion was accompanied by other genetic alterations including mutations in *TP53*, *ATRX*, and *PTEN* [47]. This report preceded the availability of DNA methylation-based categorization for PLNTY, so it is not known from the published report if the primary or recurrent tumor aligned to PLNTY by epigenetic analysis. Molecular-pathological correlation could help to identify such rare, clinically aggressive examples of tumors that align with PLNTY by histology, which require further study.

## Introduction of Pediatric-Type Diffuse High-Grade Gliomas

The pediatric-type diffuse high-grade gliomas now comprise four distinct types, two of which are new entities. Because the diagnostic term “glioblastoma” now refers to adult-type isocitrate dehydrogenase (IDH) and H3-wildtype diffuse high-grade gliomas, it is no longer recommended terminology in pediatric patients. The WHO CNS 4 diagnosis of “diffuse midline glioma, H3 K27M-mutant” has been updated to the more general nomenclature of “diffuse midline glioma, H3 K27-altered” (DMG). The naming change reflects the identification of cases of DMG that have dysregulation of H3 K27 trimethylation without the specific lysine (K) to methionine (M) substitution. The official names for histone genes were recently updated to *H3-3A*, *H3C2*, and *H3C3*, replacing the older names of *H3F3A*, *HIST1H3B*, and *HIST1H3C* [48]. Histone proteins also have a slightly different amino acid numbering system than other proteins, so in some situations the lysine position 27 and glycine position 34 are instead numbered as positions 28 and 35, respectively. The actual diagnostic terminology in WHO CNS 5 uses the nomenclature of K27 and G34, but the alternative numbering systems (p.K28 and p.G35) may be encountered in molecular reports that refer to the gene transcript, scientific literature, and other contexts [48].

**Fig. 5:**
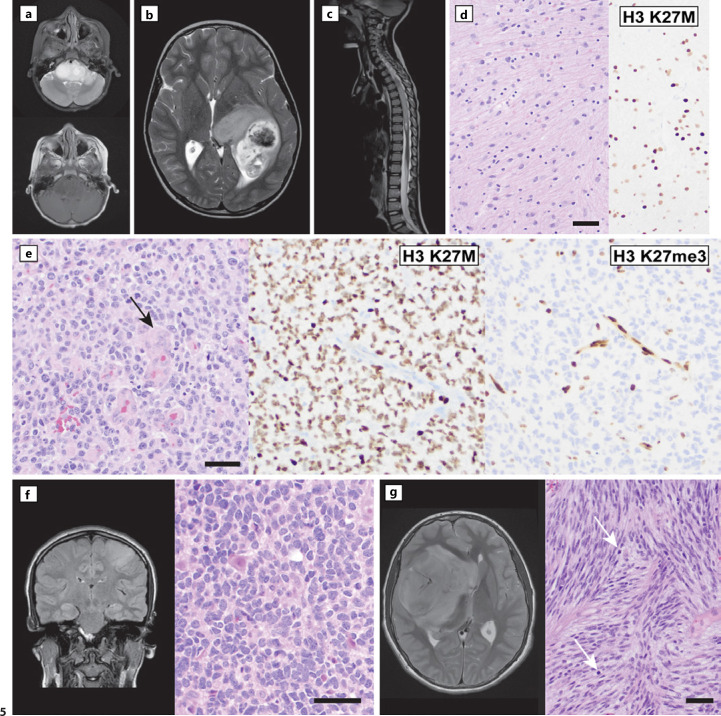
Pediatric-type diffuse high-grade gliomas. **a–e** Examples of diffuse midline glioma, H3 K27-altered. Tumor locations can range from (**a**) the prototypical clinically and radiologically defined diffuse intrinsic pontine glioma expansile T2-hyperintense (top), non-enhancing (bottom, T1 post-contrast) brain stem mass, to lesions involving the thalamus (**b**, T2) or spinal cord (**c**, T2). **d** Histology from the case shown in panel (**a**) revealed an infiltrative, low-cellularity astrocytoma that lacked mitotic activity, microvascular proliferation, and necrosis in the sampled tissue. Despite the low-grade histology, positivity for H3 K27M-mutant protein (right) is diagnostic for diffuse midline glioma H3 K27-altered in this context, and the tumor is designated CNS WHO grade 4. **e** The histology corresponding to case (**b**) was overtly grade 4 by histology, with high cellularity, marked cytologic atypia, and microvascular proliferation (arrow), with subgrouping confirmed by immunoreactivity for H3 K27M (center panel). These tumors show loss of trimethylated H3 K27 (right, positive nuclei are non-neoplastic endothelial cells) which can support subtyping in cases that have H3 K27 alterations other than the K27M missense mutation, such as EZHIP overexpression or *EGFR* mutation (see main text). Note that the PFA type of posterior fossa ependymoma also shows loss of H3 K27me3 (**f**), and proper histologic context is needed for either diagnosis. **f** Example of diffuse hemispheric glioma, H3 G34-mutant, CNS WHO grade 4, presenting as a frontal mass in a 16-year-old female. This tumor overlapped histologically with CNS embryonal tumors, showing poorly differentiated infiltrative cells with high nuclear to cytoplasmic ratios and nuclear molding. Lack of reactivity for the glial markers OLIG2 and GFAP (not shown) can present a diagnostic challenge. An *H3-3A* G34R (p.G35R) mutation was confirmed by DNA sequencing in this example. **g** This case of pediatric-type diffuse high-grade glioma, H3-wildtype, and IDH-wildtype occurred in a 12-year-old boy presenting with worsening headaches and a left-sided facial droop. Neuroimaging showed a large, T2-hyperintense mass causing midline shift. Histology showed a high-grade glioma (arrows denote mitotic figures) with spindled morphology. Genetic studies were negative for *IDH1*, *IDH2*, and H3 gene mutations. Classification by DNA methylation-based profiling identified this tumor as diffuse pediatric-type high-grade glioma, *MYCN*-amplified. All scale bars, 50 microns.

Examples of diffuse midline glioma, H3 K27-altered (CNS WHO grade 4) are shown in Figure 5a-c. The tumor histomorphology can range from mildly atypical, low-cellularity infiltrating astrocytic cells to frank high-grade histology, with microvascular proliferation, mitotic activity, and necrosis (Fig. 5d, e). A mutation-specific antibody for the H3 K27M-mutant protein can be used to support the diagnosis in small biopsies and performs well in samples with low cellularity, two issues that may arise because of the sensitive location of these tumors and potential morbidity of extensive tissue sampling. Antibody positivity does not determine which histone H3 isoform is mutated, and sequencing studies are required to identify the mutated gene (i.e., *H3-3A*, *H3C2*, *H3C3*). Tissue sampling is especially important in cases with atypical imaging features; such tumors that present clinically/radiologically as atypical diffuse intrinsic pontine glioma can represent pathologies other than DMG [49]. In addition to the H3 K27-mutant cases with mutations in the canonical (H3.1 or H3.2) or non-canonical (H3.3) variants, two additional molecular subtypes of DMG are overexpression of EZHIP protein and *EGFR* pathogenic mutation. These are included under the entity of diffuse midline glioma because, like the H3 K27M-mutant tumors, DMG with EZHIP overexpression or *EGFR* mutations also show evidence for loss of H3 K27 trimethylation. *EGFR*-mutant DMG is rare but notable for an enrichment of cases with hypothalamic (often bilateral) involvement and the occurrence of small in-frame insertions/duplications within EGFR exon 20, driving downstream pathway activation by a mechanism that could be responsive to targeted inhibition [50, 51]. Rarely, nondiffuse glial/glioneuronal tumors aligning to PA or ganglioglioma and other neuroepithelial tumors are found to have H3 K27M mutation [48]. These rare cases require further study and reinforce the requirement for a diffuse growth pattern by histology and a midline anatomical location in the diagnosis of DMG [1, 52].

The new entity of diffuse hemispheric glioma, H3 G34-mutant (CNS WHO grade 4), was originally identified in large-scale sequencing studies of non-midline high-grade gliomas in children and adolescents [53]. These are infiltrative tumors involving the cerebral hemispheres in older children and young adults, with a median age at presentation of approximately 19 years and a slight male predominance [54]. They are defined genetically by *H3-3A* gene mutations leading to a glycine (G) to arginine (R) or valine (V) substitution at amino acid position p.G35 (denoted G34 R/V − see nomenclature note above). Histologically, tumors can have astrocytic cytologic features and immunoreactivity for glial markers, or a “small round blue cell” embryonal morphology with loss of glial marker immunoreactivity (example case shown in Fig. 5f). No difference has been identified in the clinical behavior, demographics, or genetic/epigenetic features of these two histologic patterns [54]. The diagnosis is supported by demonstration of an *H3-3A* mutation by DNA sequencing or by DNA methylation-based profiling (note that mutations of *H3C2* and *H3C3* have not been identified in diffuse hemispheric glioma to date). Mutation-specific antibodies are employed at a few academic institutions, but the availability of DNA sequencing and low sensitivity and specificity of the antibodies have precluded widespread adoption [55]. Due to the range of histologic patterning from glial to embryonal and the need for molecular profiling to confirm the tumor subtyping, diffuse hemispheric glioma H3 G34-mutant should be considered in the differential diagnosis for hemispheric high-grade gliomas or supratentorial CNS embryonal tumors, especially in older children and adolescents.

Infant-type hemispheric glioma is a high-grade hemispheric astrocytic glioma of early childhood. Two large study cohorts showed that receptor tyrosine kinase fusions are important recurrent drivers in this age group including *ALK*, *MET*, *ROS1*, and the *NTRK* gene family [56, 57]. Although clinical data are limited, there are reports of favorable treatment responses to targeted therapy with tyrosine kinase inhibitors [56, 58]. Neurosurgical intervention may be indicated to acquire diagnostic tissue for molecular testing to enable targeted therapy and to exclude other forms of infant/perinatal intracranial malignancy [59]. The histological and clinical spectrum of *NTRK, ALK*, *RET*, and *ROS1* rearranged tumors requires further study.

The fourth entity in this category is “diffuse pediatric-type high-grade glioma, H3-wildtype, and IDH-wildtype” (example in Fig. 5g). This is defined as a histologically malignant (i.e., mitotically active, with or without microvascular proliferation or necrosis) diffuse glioma occurring in childhood, adolescence, or young adulthood that is proven to lack mutations of *IDH1*, *IDH2*, and H3 encoding genes. Thus, this diagnosis relies heavily on genetic, demographic, and histological correlations to exclude the adult-type IDH mutant diffuse gliomas and the pediatric-type H3 K27-altered high-grade gliomas described above. Three epigenetic subtypes can be resolved by DNA-MP, and recurrent genetic drivers include oncogene amplifications such as *PDGFRA*, *EGFR*, and *MYCN* [60].

## Updates to Embryonal Tumors of the CNS

The CNS embryonal tumors encompass high-grade, poorly differentiated neoplasms that may only be classifiable after extensive histological and genetic studies. These tumors present a formidable diagnostic and clinical challenge, and at the time of intraoperative consultation, many of them can only be characterized descriptively as “small round blue cell” tumors. Any use of the obsolete nomenclature of “CNS primitive neuroectodermal tumor (CNS PNET)” is discouraged, as this has now been proven to be an unspecific category encompassing about two dozen distinct entities [61]. WHO CNS 5 diagnostic terminology of “CNS embryonal tumor, NOS” or “NEC” is applied in the setting of a primary CNS tumor with embryonal histology that is unclassified due to lack of molecular testing or failure to identify a specific alteration after thorough genetic testing. It is presumed that the NEC tumors are mixed group of rare, poorly understood neoplasms, potentially with novel/unidentified genetic drivers, that cannot be reliably separated into CNS WHO grades 3 or 4 due to their heterogeneity and very limited outcome data.

**Fig. 6:**
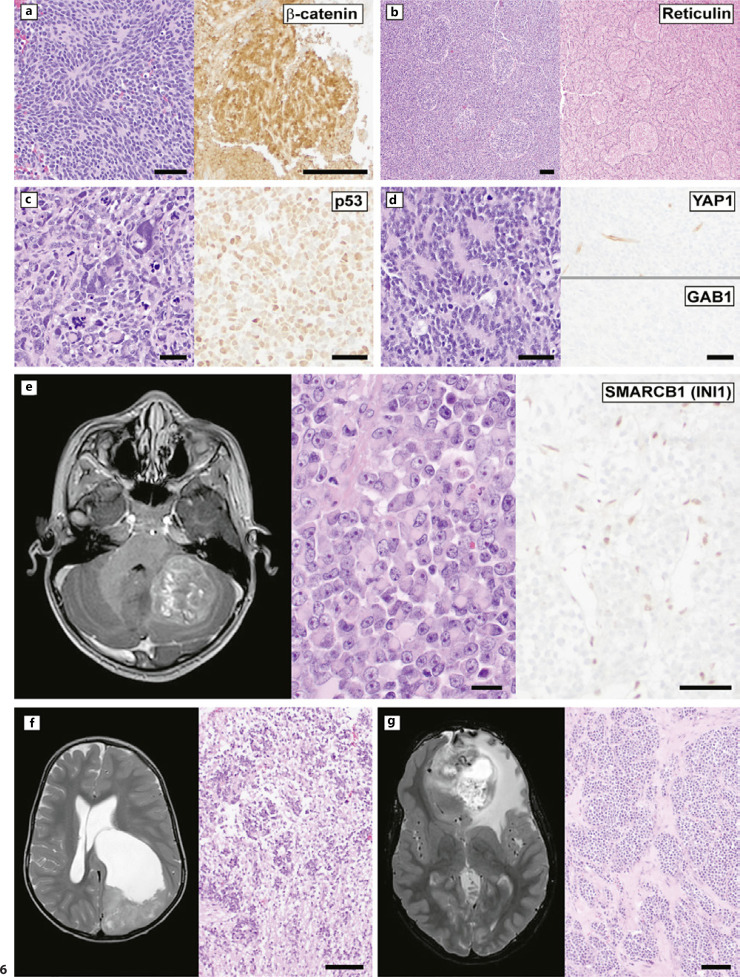
Pediatric CNS embryonal tumors. **a–d** Examples of medulloblastoma subtypes, with key pathological markers. **a** This group 1 (WNT) medulloblastoma showed classical histology and nuclear staining for β-catenin. **b** Group 2 (SHH) medulloblastomas can have nodularity on H&E staining, with reticulin histochemistry revealing reticulin-free nodules separated by reticulin-rich internodular desmoplastic zones, defining them histologically as nodular/desmoplastic or, in extreme form in infants, medulloblastoma with extensive nodularity. **c** This large cell/anaplastic medulloblastoma was subtyped as group 2 (SHH) based on molecular studies, with diffuse nuclear staining for p53 supporting the presence of a *TP53* mutation. **d** The non-WNT/non-SHH (group 3/4) subtype has classical histology in most cases. This subtype can be differentiated from medulloblastoma groups 1 and 2 by lack of reactivity for YAP11 and GAB1 immunohistochemical stains. **e–g** Examples of non-medulloblastoma CNS embryonal tumors. **e** Atypical teratoid/rhabdoid tumor presenting as a heterogeneously enhancing, well-circumscribed posterior fossa mass in a 4 year-old boy (T1-weighted post-contrast imaging). Histology showed a focally dyscohesive malignancy with tumor cells showing very large nuclei containing large, bright nucleoli. The tumor cells have rhabdoid features, represented by the eccentric nucleus and plump belly of brightly eosinophilic cytoplasm. Loss of nuclear reactivity for SMARCB1 (INI1) in tumor cells confirms the diagnosis. **f** Embryonal tumor with multilayered rosettes is a rare entity, presenting in this example as a rapidly growing parieto-occipital cystic mass in a 2-year-old boy (T2-weighted imaging). Histology shows foci of perivascular rosettes composed of poorly differentiated, high nuclear to cytoplasmic ratio embryonal cells separated by zones of lower cellularity, eosinophilic, fibrillary neuropil. **g** Radiology (T2-FLAIR) and histology of CNS neuroblastoma, *FOXR2*-activated, presenting as a complex bifrontal mass in an 11-year-old female who presented with diplopia, headaches, and behavioral changes. Histology showed nests of poorly differentiated cells separated by fibrous septae, while other areas (not pictured) appeared diffusely infiltrative through background brain. Scale bars, 50 microns (**a, c, d**), 20 microns (**e**), and 100 microns (**b, f, g**).

The existing embryonal tumor subtypes of medulloblastoma, atypical teratoid/rhabdoid tumor, and embryonal tumor with multilayered rosettes are maintained from the prior WHO classification. Medulloblastomas are classified into four histological subtypes and four molecular subtypes, as shown as examples in Figure 6a-d. The correlations between histological and molecular subtypes have been recently reviewed elsewhere [62]. Genetic and epigenetic analysis supports that there are many more molecularly distinct medulloblastoma subtypes, including about 8 subclasses within the non-WNT/non-SHH (group 3/4) tumors [63]. Future updates to the WHO are likely to incorporate new clinically meaningful medulloblastoma subtypes as diagnostic biomarkers are identified and with the increased use of molecular profiling. For example, DNA-MP has identified two molecular groups within infant SHH medulloblastoma which differ in their progression-free survival [64]. Many medulloblastoma clinical trials have an enrollment cutoff within 30 days of surgery and require comprehensive molecular profiling for enrollment. Clear communication between the treating clinician, surgeon, and neuropathologist is required to manage tissue samples for clinical and research trial requirements and to achieve a timely diagnosis and complete molecular workup. The complex relationship between medulloblastoma pathology, molecular correlation, and clinical management is beyond the scope of this review but has been recently described in detail elsewhere [5].

There is evidence that atypical teratoid/rhabdoid tumor (AT/RT) comprises three epigenetically distinct subtypes designated AT/RT-SHH, AT/RT-TYR, and AT/RT-MYC which differ in clinical presentation, histologic appearance, and epidemiology, and can be resolved by DNA-MP or gene expression profiling [65]. Tumor cell loss of expression of SMARCB1 protein (also called INI1), as shown in the example in Figure 6f, or (rarely) loss of SMARCA4 protein (also called BRG1) predicts mutations of *SMARCB1* or *SMARCA4*, respectively, with *SMARCA4*-mutant AT/RT usually presenting at younger age. Histological biomarkers for AT/RT subgrouping are an active area of research. Embryonal tumor with multilayered rosettes (ETMR, Fig. 6f) is a rare, usually supratentorial embryonal tumor with a very poor prognosis. It is defined genetically by alterations of a microRNA cluster on chromosome 19 (C19MC) or, in rare cases, alterations of *DICER1*. ETMR encompasses three histological patterns: embryonal tumor with abundant neuropil and true rosettes, medulloepithelioma, and ependymoblastoma.

CNS neuroblastoma, *FOXR2*-activated (CNS WHO grade 4), is a newly recognized tumor that was identified in 2016 through epigenetic subgrouping of histologically unclassifiable supratentorial CNS embryonal tumors (example in Fig. 6g) [61]. The tumor can occur in all pediatric age groups and rarely over age 20, and typically are located in cerebral hemispheres [61, 66]. Radiologically, CNS neuroblastoma *FOXR2*-activated appears as a demarcated mass which may have a cystic component and show contrast enhancement. Morphologic and immunohistochemical studies suggest features of neuronal differentiation along with expression of OLIG2 in most cases, and these tumors may show infiltration of CNS parenchyma at the histologic level [67]. Diagnostic criteria require evidence of *FOXR2* structural rearrangement or a compatible DNA methylation-based categorization. In one recent study, this entity accounted for 25% of supratentorial CNS embryonal tumors [68]. Prognostic information is limited at this time, though one retrospective international study showed 5-year progression-free survival of 63% and overall survival 85% in 63 patients with available clinical follow-up after treatments including craniospinal irradiation and/or chemotherapy [66].

CNS tumors with *BCOR* internal tandem duplication are another newly recognized embryonal tumor subtype. These tend to occur in young children, with one study of 10 cases showing mean age of 3.5 years [69]. Locations are typically in the cerebrum or cerebellum and typical imaging shows large solid masses with variable levels of contrast enhancement [61, 69]. The diagnostic criteria are a primary CNS tumor with solid growth pattern, uniform oval or spindle-shaped cells with round to oval nuclei, and a dense capillary network. The characteristic genetic feature is internal tandem duplication (ITD) in exon 15 of *BCOR* which encodes a transcriptional corepressor. The same *BCOR* ITD is also seen in rare extracranial neoplasms of the kidney and soft tissue. Confirmation of this alteration is an essential criterion for the diagnosis, and a supportive DNA methylation-based profile can be helpful in challenging cases. This tumor is currently not graded due to limited information, but early data suggest poor prognosis and the potential for late recurrence [61]. An *EP300-BCOR* fusion has been reported in rare pediatric CNS tumors and appears to define a distinct subtype from the BCOR ITD [70]. These require recognition and further study and may be introduced as a new subtype in future WHO classifications.

### Other Rare Newly Recognized Tumor Types

Some of the newly introduced tumor types in WHO CNS 5 are very rare and are still being characterized. It is expected that future updates to the WHO will address the gaps in knowledge for their epidemiology, histological features, molecular underpinnings, and prognosis. Cribriform neuroepithelial tumor is a rare embryonal tumor characterized by loss of SMARCB1/INI1 but distinct from AT/RT by epigenetic features and clinical behavior [71]. Desmoplastic myxoid tumor of the pineal region, *SMARCB1*-mutant has only 7 reported cases and enters the differential diagnosis for adolescent and adult pineal tumors with desmoplasia and myxoid change [72]. Pituitary blastoma is a sellar region embryonal neoplasm occurring at a median age of 9 months associated with germline *DICER1* mutations [73]. Primary intracranial sarcoma, *DICER1*-mutated, can be another manifestation of germline *DICER1* syndrome and is also seen sporadically or in the context of neurofibromatosis type 1. This tumor enters the differential diagnosis of pediatric intracranial malignant mesenchymal neoplasms, including cases with myogenic and/or cartilaginous differentiation [74]. Intracranial tumors with *CIC* rearrangements can also have neuroepithelial or mesenchymal features; this is a provisional entity in WHO CNS 5, and it is debated whether these represent primary neuroepithelial tumors or sarcomas [1].

There is increasing recognition of CNS tumors that are driven by recurrent gene rearrangements that can cross clinical, demographic, and histological boundaries. Examples include brain and spine tumors with neurotrophic tyrosine receptor kinase gene rearrangements (encoded by *NTRK1/2/3*), and tumors with rearrangements involving the *PATZ1* gene [75, 76]. Tumors with *NTRK* gene rearrangements can occur well beyond infancy and childhood and show a stunning array of histological patterns ranging from infiltrative astrocytic or oligodendroglial tumors to circumscribed gliomas and glioneuronal tumors with low- or high-grade histological features [76]. Epigenetic studies have not identified a unifying DNA methylation-based subgrouping for NTRK fusion-positive CNS tumors, suggesting that differences in cell of origin, timing of oncogenesis during development, and/or co-occurring genetic alterations contribute to their diversity. Such rare and challenging tumors might not fall into a precise category, even with molecular profiling. Gene rearrangements can also drive pediatric intracranial non-neuroepithelial CNS tumors, with the recently discovered entity of intracranial mesenchymal tumor *FET-CREB* fusion positive being one example [1]. Precisely defined fusion-driven molecular entities may be introduced in future versions of the WHO CNS.

## Conclusion

The complexity and nuance in pediatric CNS tumor diagnosis have expanded with the new entities and more sophisticated tumor typing in the recent WHO classification updates. Open communication between pathologists and clinicians is required to give clarity to tumor diagnosis and ensure the most appropriate treatment for patients. Molecular information from tumors can refine a diagnosis, define clinical trial eligibility, and reveal molecular targets for therapeutic intervention, but it also introduces challenges for timing of a final diagnosis, potential need for extramural or centralized molecular testing, and broadening of a purely histological differential diagnosis. Updates to prognostic information, clarity on tumor grades, and new or refined diagnostic categories are expected as rare tumor types become more readily identified and shared across institutions. The development of molecular and tissue markers for more rapid subclassification and better prognostication is expected to guide future revisions to the CNS WHO classification, presenting an opportunity for collaborative, multidisciplinary research efforts.

## Conflict of Interest Statement

The authors have no conflicts of interest to declare.

## Funding Sources

No funding was received in support of this work.

## Author Contributions

Dr. Aaron M. Halfpenny and Dr. Matthew D. Wood identified topics, reviewed literature, prepared the figures, and wrote the manuscript. Both authors reviewed the final version of the manuscript.

## Figures and Tables

**Table 1:** Selected examples of layered diagnostic reporting

Tumor category	Key clinical and pathological findings	Layered reporting example
Pediatric-type diffuse high-grade gliomas	Diffusely infiltrative astrocytic glioma, midline (brain stem) location, positive for histone H3 p.K28M (K27M) by immunohistochemistry	Integrated diagnosis: diffuse midline glioma, H3 K27-altered, CNS WHO grade 4 Histological classification: diffuse high-grade astrocytoma with microvascular proliferation and necrosis CNS WHO grade: 4 Molecular information: positive for histone H3 K27M-mutant protein by immunohistochemistry

Pediatric-type diffuse high-grade gliomas	Diffuse glioma with embryonal features, hemispheric location, adolescent patient, positive for *H3-3A* p.G35R or p.G35V (G34 R/V) by DNA sequencing	Integrated diagnosis: diffuse hemispheric glioma, H3 G34-mutant, CNS WHO grade 4 Histological classification: diffuse high-grade astrocytoma with embryonal features CNS WHO grade: 4 Molecular information: positive for H3-3A p.G35R mutation by DNA sequencing. Positive for ATRX and TP53 gene mutations by immunohistochemistry and sequencing. MGMT promoter methylation status: hypermethylated

Embryonal tumors	Cerebellar mass, compact/non-infiltrative embryonal tumor with nodular regions and increased internodular reticulin, immunoreactive for synaptophysin, YAP1, and GAB1, retained INI1 protein, low tumor cell labeling with p53 immunostain	Integrated diagnosis: medulloblastoma, SHH-activated and TP53-wildtype, CNS WHO grade 4 Histological classification: desmoplastic/nodular medulloblastoma CNS WHO grade: 4 Molecular information: immunohistochemistry positive YAP1 and GAB1. Low p53 labeling, consistent with wildtype *TP53.* Negative for *MYC/MYCN* amplification by FISH

Embryonal tumors	Supratentorial mass in an infant, malignant spindled, epithelioid, and/or embryonal neoplasm with rhabdoid cells, loss of INI1 protein by immunohistochemistry	Integrated diagnosis: atypical teratoid rhabdoid tumor, CNS WHO grade 4 Histological classification: malignant rhabdoid tumor CNS WHO grade: 4 Molecular information: negative for INI1 by immunohistochemistry

Pediatric-type diffuse low-grade glioma	Non-enhancing, poorly demarcated, expansile cerebral hemispheric mass, long history of refractory seizures, mildly atypical infiltrative astrocytic cells without mitotic activity, necrosis, or vascular proliferation, DNA sequencing positive for BRAF p.V600E mutation	Integrated diagnosis: diffuse low-grade glioma, MAPK pathway-altered Histological classification: diffusely infiltrative low-grade astrocytoma CNS WHO grade: not assigned. Behavior is predicted to correspond to CNS WHO grade 1 to 2 Molecular information: positive for BRAF p.V600E by DNA sequencing

**Table 2:** Summary of the WHO CNS 5 updates discussed in this review

*Prior nomenclature or ‡previous general category^1^	WHO CNS 5 classification	Comments
*Diffuse midline glioma, H3 K27M-mutant	Diffuse midline glioma, H3 K27-altered	Widens the spectrum of H3 K27 alterations beyond K27M mutation^2^

*Supratentorial ependymoma, *RELA* fusion positive	Supratentorial ependymoma*, ZFTA* fusion positive	Updates the relevant fusion partner driver to *ZFTA* and updates gene nomenclature

*Astroblastoma	Astroblastoma, *MN1*-altered	Adds genetic qualifier

*Hemangiopericytoma	Solitary fibrous tumor	Aligns to systemic nomenclature, unifies two entities into one

*Myxopapillary ependymoma, WHO grade I	Myxopapillary ependymoma, CNS WHO grade 2	Updated to CNS WHO grade 2

‡Glioblastoma and anaplastic astrocytoma (pediatric)	Diffuse pediatric-type high-grade glioma, with subtype^3^	Reflects more precise criteria for glioblastoma diagnosis (usually adult patients), and recognizes subtypes in pediatric diffuse high-grade gliomas

‡Supratentorial ependymoma	Supratentorial ependymoma, *YAP 1* fusion positive	Rare, younger age at presentation, female predominance, potential better outcome than *ZFTA*

‡Ependymoma (posterior fossa)	Posterior fossa ependymoma group A or B (PFA/PFB)	PFA is clinically aggressive, identifiable by genetic/epigenetic features and can be suggested by a histological surrogate marker

‡Low-grade glial/glioneuronal tumor such as DNET or ganglioglioma	MGNT	Epilepsy associated, location at septum pellucidum or periventricular, *PDGFRA* p.K385 alteration

‡Low-grade glial/glioneuronal tumor	MVNT	Distinctive radiology, longstanding epilepsy may arise in childhood, most resections are in adulthood

‡Low-grade or high-grade, circumscribed or diffuse, glial, or glioneuronal tumor	DGONC	Rare, diagnosis requires genome-wide DNA methylation-based profiling, often monosomy 14, molecular driver unknown

‡Astrocytic glioma (appearing either low-grade or high-grade)	HGAP	Rare, diagnosis requires genome-wide DNA methylation-based profiling, frequent MAPK pathway, *CDKN2A/B,* and *ATRX* alterations

‡Glioblastoma or unspecified CNS embryonal tumor	Diffuse hemispheric glioma, H3 G34-mutant	Histology can mimic a supratentorial CNS embryonal tumor

‡High-grade astrocytoma	Infant-type hemispheric glioma	Possible response to targeted therapy

‡Unspecified CNS embryonal tumor	CNS neuroblastoma, *FOXR2*-activated	Accounts for a substantial proportion of supratentorial CNS embryonal tumors

‡Low-grade glial/glioneuronal tumor with oligodendroglial morphology	PLNTY	Epilepsy associated, strong CD34 immunoreactivity

‡Diffuse low-grade astrocytoma	Diffuse astrocytoma, *MYB* or *MYBL1* altered	Epilepsy associated, infiltrating astrocytic low-grade glioma, rare cases reported in brain stem location (potential mimic of DIPG)

DNET, dysembryoplastic neuroepithelial tumor.

1 General tumor category is meant to describe what diagnostic categories these new entities may have fallen into, before they were recognized and separated out as a distinct entity.

2 See text for note on histone amino add numbering and gene nomenclature.

3 Subtype may include diffuse midline glioma, H3 K27-altered, listed above.
